# Legal Barriers to Alcohol Screening in Emergency Departments and Trauma Centers

**Published:** 2004

**Authors:** Linda Chezem

**Affiliations:** Linda Chezem, J.D., is an assistant to the Director, National Institute on Alcohol Abuse and Alcoholism, Bethesda, Maryland. She also is a professor in the Department of Youth Development and Agricultural Education, Purdue University School of Agriculture, in West Lafayette, Indiana, and an adjunct professor with the Indiana University School of Medicine in Indianapolis, Indiana. She is a retired appeals court and circuit court judge

As described in more detail in the accompanying article by D’Onofrio and Degutis, many patients admitted to emergency departments (EDs) and trauma centers have positive blood alcohol levels at the time of their visit. (For more information on the distinction between EDs and trauma centers and the patients they treat, see the textbox “Emergency Departments Versus Trauma Centers.”) Research has shown that screening ED and trauma patients for alcohol use not only helps physicians make a more accurate diagnosis of patients’ conditions and decide on an appropriate treatment plans but also may allow for brief interventions and referrals to more extensive treatment. Many clinicians believe that patients with alcohol-related problems may be particularly amenable to alcohol interventions while they receive acute medical care for an alcohol-related injury. Several studies have demonstrated that brief interventions delivered to patients who are being treated in EDs or trauma centers for alcohol-related injuries can reduce alcohol consumption and the risk of renewed alcohol-related injuries in those patients (for more information, see the article by D’Onofrio and Degutis).

**Emergency Departments Versus Trauma Centers**

The emergency department (ED) is the division of a hospital that provides care to patients with sudden and acute illnesses or injuries. EDs treat patients with all kinds of medical conditions, including people who have no health insurance or primary care physician and therefore use the ED as their only source of health care. Thus, both the types and the severity of the conditions treated cover a very broad spectrum.

Trauma centers, in contrast, only treat patients with severe physical injuries, such as wounds, burns, or fractures, many of which require surgery by a specialized trauma surgeon. As a result, the spectrum of conditions treated at a trauma center is narrower than in an ED, whereas the condition of most trauma center patients is more serious than that of the average ED patient. In many hospitals in small or mid-size towns or rural areas, however, at least initial care to trauma patients typically is provided in EDs.

Both EDs and trauma centers treat patients with alcohol-related conditions. Trauma centers primarily see patients with acute alcohol-related problems, and EDs see problems related to long-term alcohol use.

Despite the apparent benefits of screening and brief interventions or referrals, only a portion of ED and trauma patients actually are screened for alcohol use and alcohol-related problems. One survey found that about two-thirds of trauma surgeons frequently determine the blood alcohol concentrations of their patients, but only 25 percent used formal screening questionnaires with some or all of their patients (Schermer et al. 2003). Thus, although the study’s authors noted an increase in screening over previous years, a large number of patients who could benefit from screening still are missed. It is important to note, however, that this survey was conducted among trauma surgeons, who are more likely to see severely injured patients than are ED physicians, who see injured patients as well as patients with a broad range of other medical problems of varying severity. Therefore, the findings of this survey may not entirely reflect the actual prevalence of screening in ED patients or the frequency of screening in EDs that treat trauma patients because there is no dedicated trauma center in the area.

In any event, the question remains why all trauma and ED health care professionals are not screening all their patients for possible alcohol problems. Several factors have been suggested as potential barriers to screening, including professionals’ doubts concerning the effectiveness of interventions for alcoholism, lack of time and resources to conduct screening, increased health care costs, and concerns about patient confidentiality. In addition, health care providers may fear that because of existing laws, third-party payors (i.e., insurers) may deny reimbursement for medical services if a patient has a positive blood alcohol level at the time of the ED visit. Some observers have identified the legal provisions that deal with alcohol use and the insurance payment of benefits for medical care as a factor that may contribute to the failure of many medical care facilities, particularly EDs, to screen for alcohol abuse and dependence as well as other alcohol-related problems. For example, the previously mentioned survey among trauma surgeons found that 27 percent of the respondents felt that screening would threaten reimbursement of medical costs (Schermer et al. 2003). The following discussion explores this issue in more detail.

## Insurance Laws and Reimbursement of Alcohol-Related Medical Claims

In the United States, the provision of health care benefits by insurance companies is considered one of the powers that the Tenth Amendment reserves to the individual States because it is not specifically listed as a prerogative of the Federal Government. (For more information, see the textbox “History of State Versus Federal Legislative Authority.”) One organization that informs much of the States’ regulatory and legislative work regarding the insurance industry is the National Association of Insurance Commissioners (NAIC), an organization of insurance regulators from the 50 States, the District of Columbia, and the four U.S. territories. The NAIC was established in 1871 to support and coordinate the work of State regulatory bodies.

**History of State Versus Federal Legislative Authority**

The treatment of alcohol-related conditions and actions by the law has been influenced by legal history regarding the States’ powers. The Tenth Amendment reserves to the individual States all powers not specifically listed as powers of the Federal Government. In the case of insurance law, this is explicitly recognized and reinforced by the 1945 McCarran-Ferguson Act, which in Section 1011 states: *“Declaration of policy—Congress hereby declares that the continued regulation and taxation by the several States of the business of insurance is in the public interest, and that silence on the part of the Congress shall not be construed to impose any barrier to the regulation or taxation of such business by the several States”* (Act Mar. 9, 1945, ch. 20, 59 Stat. 33).

As a result of this division of powers, the States maintain their independent roles and can draw up a variety of laws and regulatory policies, allowing for testing the laws’ efficacy and applicability to the specific needs of their citizens. The States’ legislative independence also can result in policy and legislative advances (e.g., the repeal of UPPL statutes) that are easier to achieve on the State level than they would be on the Federal level.

In 1947, the NAIC developed a model code entitled the Uniform Accident and Sickness Policy Provision Law (UPPL), which has left a legacy that still discourages medical providers from screening patients for alcohol misuse and intervening with people who have alcohol abuse and dependence or other alcohol-related problems. The UPPL is rooted in centuries-old English common law, which considers alcohol-related acts and conditions evidence of moral failure that should be punished. Reflecting this view (and the lack of modern alcohol science), the UPPL included language that allowed insurance carriers to deny benefits if the injury or condition were “sustained or contracted in consequence of the insured’s being intoxicated or under the influence of any narcotic unless administered on the advice of a physician” (NAIC 1947).

**At a Glance**

**Legal Barriers to Alcohol Screening in Emergency Departments and Trauma Centers**

- About two-thirds of trauma surgeons frequently assess patients’ blood alcohol concentrations (Schermer et al. 2003).
- 25 percent of trauma surgeons used formal screening questionnaires with some or all their patients (Schermer et al. 2003).
- 27.7 percent of trauma surgeons felt that screening would threaten reimbursement of medical costs (Schermer et al. 2003).
- 38 States and the District of Columbia had Uniform Accident and Sickness Policy Provision Law (UPPL) provisions in their insurance laws in 2000 allowing insurers to deny insurance coverage for injuries sustained while a patient was intoxicated or under the influence of another drug (Rivara et al. 2000).
- 8 States had no statutes regarding denial of coverage for alcohol-related injuries or conditions (Rivara et al. 2000).
- A few States have repealed their UPPL statutes since the National Association of Insurance Commissioners repealed the UPPL provisions in 2001 and adopted a new model law, and the National Conference of Insurance Legislators passed a resolution in support of this new model law.

A survey of all 50 States and the District of Columbia published in 2000 found that 38 States and the District of Columbia still had the UPPL provisions in their insurance codes. In these States, insurers could deny insurance coverage for injuries sustained while the patient was intoxicated or under the influence of another drug (Rivara et al. 2000). A few other States allowed insurers to deny coverage only if the patient was under the influence of narcotics (but not alcohol) or if an injury was sustained while the insured committed a felony. Only eight States had no statutes regarding the denial of coverage for alcohol-related injuries or conditions. Even in these States, however, the statutes do not expressly prohibit insurers from denying this coverage. In fact, the survey found that many of the State insurance commissioners felt that by drinking alcohol people knowingly put themselves in harm’s way, and their claims therefore could be denied, just as claims for self-inflicted injuries resulting from suicide attempts can be denied (Rivara et al. 2000).

Consistent with these attitudes toward alcohol consumption and its consequences, decisions in legal cases at the State, territorial, or Federal level are replete with descriptions of the legal roots of considering misuse of alcohol^1^ as a moral failing. In hundreds of reported cases, courts have ruled that alcohol misuse by an insured person is a legitimate reason for the denial of insurance benefits when the statutes or the applicable benefits policies exclude alcohol-related injuries or conditions.

Over the past 60 years, however, scientific research has vastly altered and enhanced our understanding of alcohol abuse and dependence so that they now are considered medical disorders much like other common disorders such as heart disease and diabetes. In recognition of these advances, the NAIC in 2001 voted to repeal the original UPPL provisions. The organization adopted a new model law that bars insurers from denying insurance benefits to patients whose injuries or conditions are alcohol related. The recommendations in this model law are in line with those published by the National Conference of Insurance Legislators (NCOIL), an organization made up of State legislators whose main area of public policy concern is insurance legislation and regulation. In March 2001, NCOIL passed a resolution in support of NAIC’s new model law. Thus, these two important and influential national organizations support the provision of benefits for alcohol-related injuries and conditions.

Despite these recommendations, however, there has been little legislative action at the State level to repeal the old laws. Only a few States, including Maryland, Vermont, and North Carolina, have repealed their UPPL statutes (California Assembly Committee on Health 2004). And in the State of Washington, House Bill 2014 became effective on June 10, 2004, prohibiting health insurers and health maintenance organizations from denying claims for treatment of an injury solely because the injury was sustained as a consequence of the insured’s being intoxicated or under the influence of narcotics. In this bill, the legislators wrote: “The legislature finds that an alcohol or drug-related injury that requires treatment in an emergency department can be a critical moment in the life of a person with a substance abuse problem. Studies have demonstrated that appropriate interventions by hospital staff at these times can reduce substance abuse and lower future health care costs. The perception among health care providers that they may be penalized by insurers for conducting these interventions prevents many of them from performing interventions which can make all the difference to a person at the crossroads of a substance abuse problem” (Rev. Code Wash. [ARCW] § 48.21.125). Thus, at least in this case policymakers appear to consider basing practices on scientific evidence to be the best approach when developing new rules.

## Consequences of Existing UPPL Statutes

In some States and with certain third-party payors, ED and trauma patients who have positive blood alcohol levels when they are injured are at risk of having their insurance benefits denied. This denial of coverage can result in a potentially enormous financial burden to the patient (e.g., if a patient requires intensive medical care after an alcohol-related car crash) or the treating hospital (if it does not receive payment from the patient or the insurer). In addition, denial of claims may extend to benefits that are not directly covered by the UPPL, such as claims for life insurance, disability insurance, workers’ compensation, and unemployment benefits. In many States, payments for workers’ compensation, as well as disability and death benefits, may be denied if the injured or deceased person had consumed alcohol before or during the incident leading to the claim. For example, Indiana statute I.C. § 22-3-2-8 regarding bars to compensation, states: “No compensation is allowed for an injury or death due to the employee’s knowingly self-inflicted injury, his intoxication, his commission of an offense, etc. . . . The burden of proof is on the defendant.” In the State of Colorado, nonmedical benefits can be reduced by 50 percent if the injured employee had a blood alcohol level at or above 0.1 percent at the time of the injury. Nevertheless, denial of reimbursement for alcohol-related health care expenses has received the most attention, because of an increased focus on screening and brief interventions efforts.

Although it is easy to determine the content of the various States’ insurance statutes, it is more difficult to determine how these statutes are applied without a thorough analysis of the health care payment market and other research. For example, unanswered questions include:

- Even in the States with UPPL statutes, a number of insurance companies do not deny payment for alcohol-related claims. What percentage of insurance companies in these States actually deny alcohol-related claims, and what is the market share of the policies under which the claims are denied?
- Are UPPL laws an actual barrier to screening in the ED or are they only *perceived* by ED physicians as a barrier?

Even if this information were available, the actual impact of UPPL statutes on the routine practices of ED and trauma services would be difficult to estimate. But no matter what the underlying reasons are, the cases reported from the various State and Federal courts are evidence that some denial of benefits occurs. Whether these cases are the tip of an iceberg or rare exceptions is not clear from a review of the case law.

## Other Legal Issues Related to Alcohol Screening in the ED

Another legal issue that may contribute to the reluctance of ED physicians to screen their patients for alcohol use is that patients can be denied benefits if they were injured during the commission of a criminal act (i.e., felonies and misdemeanors). In some States, driving while impaired (DWI) is considered a misdemeanor. Many States, however, classify DWI as a felony, especially if it leads to a crash severe enough to result in the need for medical attention.^2^ Many insurance policies exclude benefits for injuries sustained in the commission of a felony but not those resulting from a misdemeanor. Some policies, however, exclude benefits for injuries sustained in the commission of any criminal act; in these cases, misdemeanor offenses such as public intoxication or illegal consumption of an alcoholic beverage also could be used to justify denial of benefits.

The threat of criminal prosecution of an intoxicated patient may deter ED physicians from screening their patients for alcohol use. Anecdotal evidence suggests that this reluctance to screen stems less from a desire to protect patients from prosecution than from the medical professionals’ frustration with and intimidation by the legal requirements of dealing with alcohol-related injuries. For example, the costs in money, time, and energy can be high for ED or other care providers, who must hire counsel and respond to interrogatories, subpoenas, and other requests for documents and testimony in the event of criminal charges or civil litigation. The actual burden of justice system requests on EDs, as well as the perceived burden, still need to be determined.

## Conclusions and Outlook

Interest in screening and brief intervention for alcohol problems by ED physicians and other health care providers has remained high in recent years, as demonstrated by several research efforts to maximize the efficacy of the practices, several recent published guides, and other activities:

- The National Institute on Alcohol Abuse and Alcoholism (NIAAA) and the Substance Abuse and Mental Health Services Administration (SAMHSA) announced a major collaborative study that will investigate ways to screen, identify, and treat patients in hospital EDs for alcohol problems.
- NIAAA has developed a guide for health care providers, titled *Helping Patients Who Drink Too Much: A Clinician’s Guide*, to assist physicians, nurses, and other health care professionals in screening patients for alcohol problems and conducting brief interventions for those problems.
- The National Highway Traffic Safety Administration (NHTSA) has given a high priority to improving the understanding and implementation of screening and brief interventions in EDs across the United States. A NHTSA panel recommended that ED physicians incorporate screening for alcohol use problems into routine care of injured patients (Runge et al. 2001).
- NIAAA, NHTSA, SAMHSA, the Agency for Healthcare Research and Quality, and the Centers for Disease Control and Prevention sponsored a conference on screening and intervention in EDs in 2001 and a similar conference focusing on trauma centers in 2003.
- SAMHSA has provided funding to seven States to support screening, brief intervention, and referral projects, some of which focused on EDs.
- The American College of Emergency Physicians provides materials on screening and brief interventions for alcohol problems among ED patients on its Web site.

Despite this significant attention to screening and brief intervention in EDs and trauma centers, disconnects between existing insurance laws, current medical practices, and research advances regarding alcohol abuse and its treatment still influence the provision of these services. By bringing together the fields of law, medicine, and alcohol research, better approaches can be developed to ensure that patients are systematically and adequately screened in EDs without compromising their access to health insurance.

## Footnotes

1 The term “alcohol misuse” here refers to any use of alcohol that leads to harmful consequences (e.g., intoxication). regardless of whether it meets the clinical definitions of alcohol abuse or dependence.

2 The classification of DWI offenses depends entirely upon the law of each State. A number of States classify DWI offenses as felonies if they are repeat offenses, cause death or serious bodily injury, or involve a blood alcohol concentration over 0.15 percent, or if the person has a history of previous traffic offenses.
